# Quality of Professional Life and Burnout of the Nursing Staff at an Intensive Care Unit in Venezuela

**DOI:** 10.17533/udea.iee.v39n2e08

**Published:** 2021-06-15

**Authors:** Pedro José Quijada-Martínez, Irmarys Rosangel Cedeño-Idrogo, Guillermo Terán-Ángel

**Affiliations:** 1 Specialist in Internal Medicine, Specialist in Intensive Care and Critical Medicine. Email: pjquijadamartinez@gmail.com. Corresponding author pjquijadamartinez@gmail.com; 2 Nurse, Specialist in the Care of the Critically Ill Patient. Email: irci3@hormail.com irci3@hormail.com; 3 Biologist, M.Sc. Professor, Universidad de Los Andes. Mérida, Venezuela. Email: guillermondi@gmail.com Universidad de Los Andes Universidad de Los Andes Mérida Venezuela guillermondi@gmail.com; 4 Intensive Care Service at Instituto Autónomo Hospital Universitario de Los Andes, Mérida (Venezuela). Instituto Autónomo Hospital Universitario de Los Andes Instituto Autónomo Hospital Universitario de Los Andes Mérida Venezuela

**Keywords:** quality of life, nursing staff, intensive care units, burnout, psychological, cross-sectional studies., calidad de vida, personal de enfermería, unidades de cuidados intensivos, agotamiento psicológico, estudios transversales., qualidade de vida, recursos humanos de enfermagem, unidades de terapia intensiva, esgotamento psicológico, estudos transversais.

## Abstract

**Objective.:**

To determine the relationship between the level of quality of professional life and the characteristics of the burnout syndrome of the nursing staff in the intensive care unit.

**Methods.:**

An analytic cross-sectional study was conducted in the intensive care unit of a public hospital in Mérida (Venezuela), with the participation of 40 nurses from a total population of 43. The Professional Quality of Life of 35 items (QoPL-35) and Maslach Burnout Inventory scales were used.

**Results.:**

Of the participants, 67.5% were professionals and 32.5% were residents, < 41 years of age (75%) and of female sex (90%)**.** The professional quality of life was regular (median = 213), the intrinsic motivation dimension was the best scored (median = 76), followed by that of workload (median = 68) and that of directive support (median = 65). The prevalence of high burnout syndrome was 22.5%; emotional exhaustion affected 75.5% of the participants and 37.5% had low personal achievement. The level of professional quality of life was related with the severity of the burnout syndrome (*p*=0.04).

**Conclusion.:**

The professional quality of life of the nurses in the ICU studied was regular and is associated with a higher risk of suffering severe burnout syndrome.

## Resumen

**Objetivo.:**

Determinar la relación entre el nivel de Calidad de Vida Profesional -CVP- y las características del síndrome de Burnout del personal de enfermería en la Unidad de Cuidados Intensivos -UCI-.

**Métodos.:**

Se realizó un estudio de corte transversal analítico en la UCI de un hospital público en Mérida (Venezuela), participaron 40 enfermeros de una población total de 43. Se emplearon las escalas Calidad de Vida Profesional de 35 ítems CVP-35 (Quality of Professional Life Questionnaire QoPL-35, en inglés) y *Maslach Burnout Inventory*.

**Resultados.:**

De los participantes 67.5% eran profesionales y 32.5% eran residentes, menores de 41 años (75%) y de sexo femenino (90%)**.** La calidad de vida profesional fue regular (mediana=213), la dimensión motivación intrínseca fue la mejor valorada (mediana=76), seguida de la de carga de trabajo (mediana=68) y la de apoyo directivo (mediana=65). La prevalencia del síndrome de Burnout alto fue de 22.5%; el agotamiento emocional afectó al 75.5% de los participantes y 37.5% tenía baja realización personal. El nivel de Calidad de Vida Profesional se relacionó con la severidad del Síndrome de Burnout (*p*=0.04).

**Conclusión.:**

La CVP de los enfermeros en la UCI estudiada es regular y se asocia a un mayor riesgo de sufrir un síndrome de Burnout severo.

## Resumo

**Objetivo.:**

Verificar a relação entre o nível de qualidade de vida profissional e as características da Síndrome de Burnout da equipe de enfermagem em unidade de terapia intensiva.

**Métodos.:**

Um estudo transversal analítico foi realizado na Unidade de Terapia Intensiva de um hospital público em Mérida (Venezuela), 40 enfermeiras participaram de uma população total de 43. As escalas de Qualidade de Vida Profissional de 35 itens CVP-35 (Quality of Professional Life QoPL-35, em inglês) e Maslach Burnout foram usados. Inventário.

**Resultados.:**

Dos participantes, 67.5% eram profissionais e 32.5% residentes, menores de 41 anos (75%) e do sexo feminino (90%). A qualidade de vida profissional foi razoável (mediana = 213), a dimensão motivação intrínseca foi a mais valorizada (mediana = 76), seguida da carga de trabalho (mediana = 68) e suporte gerencial (mediana = 65). A prevalência de síndrome de Burnout alto foi de 22.5%, exaustão emocional afetou 75.5% dos participantes e 37.5% apresentaram baixa realização pessoal. O nível de Qualidade de Vida Profissional esteve relacionado à gravidade da Síndrome de Burnout (*p*=0.04).

**Conclusão.:**

A qualidade de vida profissional dos enfermeiros da UTI estudada é regular e está associada a um maior risco de sofrer de síndrome de Burnout grave

## Introduction

Professional quality of life (QoPL) is the emotional state resulting from the interaction between the demands of work and the resources (personal and institutional) available to satisfy them.(1) A Good QoPL is the feeling of job wellbeing product of the harmony among work exigencies, the capacities to comply with the tasks assigned, and the benefits obtained in exchange for the effort invested, which permit workers their optimal development, satisfy their needs and improve their life condition.(2) Thus, the perception of QoPL involves the interaction of multiple factors; personal, social, emotional, family, and institutional that condition the relations workers establish with their work environment, determining their motivation, satisfaction, and performance,(1,2) in this sense, QoPL is comprised of three structural dimensions: workload (excessive amount of work or pressure to conduct it), intrinsic motivation (emotional factors that condition conduct in the search for personal growth) and directive support (administrative support by the institution).(3)

In areas dedicated to caring and direct interaction with people, like health institutions, it is vital to comprehend the behavior of the QoPL and its influence on the quality of services provided to patients, with emphasis on the nursing staff who, besides being the most numerous component of the health staff, due to their work dynamics are prone to emotional burnout, perceiving low QoPL, and job dissatisfaction.(4,5) In special areas and of great demand. Like the intensive care unit (ICU), the nursing staff faces particular situations and of emotional stress, like constant contact with the death and suffering of patients, besides the frequent association of a high workload, strenuous work days, excessive hours on call, job inequity, scarce directive support, little recognition of the effort and inadequate financial remuneration; conditions that potentiate the susceptibility of perceiving low QoPL, cause physical and emotional discomfort with increased incidence of diseases related with work, like the burnout syndrome (BS).(5,6)

The BS is an occupational disease(6) consequence of exposure to high and sustained levels of stress capable of overcoming the individual’s protection mechanisms of adaptation and resilience, triggering emotional fatigue (feeling emotionally exhausted), dejection, tiredness, irritability, anxiety, depression and development of negative feelings, and aversion to work. The BS is composed of three dimensions: emotional exhaustion (perception of depleting energies, anxiety, and irritability when thinking about the work), depersonalization (loss of empathy with the patients and appearance of cynicism with dehumanized treatment), and lack of personal achievement (sense of not achieving goals, feeling of stagnation); the presence of emotional exhaustion and depersonalization with low personal achievement determine the presence of the BS and determine its severity.(2)

In the nursing staff working in the ICU, it is common to find the association of low QoPL and higher severity of BS, a phenomenon that has negative consequences, like increased frequency of errors related with caring, deterioration of interpersonal relations, anxiety, emotional isolation, greater rate of resignations, diminished quality of care of patients, physical exhaustion, insomnia, depression, cynical treatment of patients, diminished job performance, lack of motivation, increased work absenteeism, consumption of alcohol and tobacco;(7) situations that compromise the safety of critically ill patients, deteriorate operations in the ICU and represent a risk to the health of nurses and patients.(2,6)

In view of the severe sequelae that accompany a low QoPL and the BS, and of the relationship that exists between both conditions, it is necessary for health institutions to have up-to-date information about their characteristics to design timely strategies to maintain optimal levels of QoPL and avoid the development of the BS.(4-6) The objective of this study was to determine the level of professional quality of life and characteristics of the burnout syndrome of nurses in an ICU in Mérida (Venezuela).

## Methods

A cross-sectional study was conducted in the intensive care unit at the *Instituto Autónomo Hospital Universitario de Los Andes* in Mérida-Venezuela from July to September 2019, with participation by 40 of the 43 nurses who constituted the total population, including professionals with academic degrees in intensive care (specialists, Master’s, diploma courses) and graduate nursing degree students in intensive care (residents). The inclusion criteria had to do with having at least three months of continuous work in the unit at the moment of the study, performing functions related with direct care of critically ill patients. Three nurses were excluded; two were in administrative assignments and one manifested the desire to not participate.

Prior to collecting the study information, a brief induction was made on the theme; informed consent was requested for participation and a 15-day limit was established to deliver an administrative form answered anonymously, containing: (1) sociodemographic data; (2) the Spanish version of the Quality Professional of Life Questionnaire (QoPL-35) instrument or Quality of Professional of Life (QoPL-35) questionnaire,(8) which was used to determine the professional quality of life of the nursing staff. This questionnaire is made up of 35 questions in a Likert scale from 1 to 10 (nothing = 1; much = 10), and evaluates three dimensions: workload (11 items), directive support (13 items), and intrinsic motivation (10 items). The sum of the scores of the 35 items is interpreted in three categories: good (246 to 350 points), regular (140 to 245 points), and poor (35 to 139 points); this questionnaire has an independent question destined to knowing the perceived professional quality of life (subjective appraisal) by each individual in a scale from 1 to 10 and interpreted through the categories: nothing = 1-2, somewhat or regular =3-4-5, a lot = 6-7-8 and much = 9-10;(8) (3) The Maslach Burnout Inventory (MBI) instrument(9) to establish the behavior of the burnout syndrome, which is a questionnaire with 22 questions grouped into three dimensions: emotional exhaustion (9 items), depersonalization (5 items), and personal achievement (8 items). It evaluates the frequency with which specific work events are perceived through Likert-type response options (never = 0; sometimes per year or less = 1, sometimes per month or less = 2, a few times per month or less = 3, sometimes per week = 4, a few times per week or less = 5, and daily = 6), according to the score obtained, three categories were established for each dimension (Table 1).

**Table 1:** Classification according to the score from each dimension from the MBI instrument(9)

Dimension	Low	Medium	High
Emotional exhaustion	≤ 17	18 to 29	≥ 30
Depersonalization	≤ 5	6 to 11	≥ 12
Personal achievement	≥ 40	34 to 39	≤ 33

The severity of the BS is established according to the severity of the dimensions that compose its fundamental nucleus (emotional exhaustion and low personal achievement). However, for the instrument’s authors, work and specially that related with direct care of patients represents in itself a risk for developing BS; which is why no category exists that excludes it in its entirety, considering that all the nurses have at least a low level of such.(9)

The quantitative data are shown through medians, interquartile range, and maximum and minimum values; qualitative data are presented in frequency tables. To establish the level of each dimension from the QoPL-35 questionnaire, a division was carried out in tertiles establishing three categories: high, medium, and low for each of them. The statistical relations were determined (bivariate analyses) through the chi squared test, considering significant a *p* value ≤0.05. The statistical analyses were performed with the SPSS program version 21 (IBM Corporation, New York, US). This study was approved by the ethics committee and competent authorities at *Instituto Autónomo Hospital Universitario de Los Andes*.

## Results

Of the 40 participants, the mean age was 35.5 years (range from 24 to 70 years), highlighting two age groups; those < 30 years of age (37.5%) and those from 31 to 40 years of age (37.5%); the female sex represented 90% of the sample; 47.5% manifested being single; 40% had no economic dependents; and 57.5% had - at the time of the survey - less than 5 years of work experience in ICU. It is highlighted that 67.5% are professionals with degrees in intensive care, 57.5% reported having only one job (in the institution where the study was conducted), and the majority of the nurses (55%) work by alternating day and night shifts (Table 2).

**Table 2:** Sociodemographic characteristics of 40 nurses working in ICU

Characteristic	Number	Percentage
Age group in years		
≤ 30	15	37.5
31 to 40	15	37.5
41 to 50	4	10
≥ 51	6	15
Sex		
Female	36	90
Male	4	10
Marital status		
Single	19	47.5
Married	11	27.5
Steady relationship	6	15
Divorced	4	10
Economic dependents*		
0	16	40
1	14	35
2	6	15
3 or more	4	10
Years of professional practice		
≤ 5	23	57.5
6 - 10	6	15
11 - 15	4	10
≥ 16	7	17.5
Academic level		
Professionals	27	67.5
Residents	13	32.5
Number of jobs		
1	23	57.5
2 or more	17	42.5
Shift		
Day	6	15
Night	12	30
Both	22	55

* Economic dependents: Persons who lack economic autonomy and depend on others for their support.

The professional quality of life of the 40 participants is regular (median = 213), the dimension of intrinsic motivation was the best scored (median = 79), followed by the dimensions of workload (median = 68) and directive support (median = 65). The perceived professional quality of life (independent question) obtained a median = 5 that is within the category of somewhat or regular (Table 3).

**Table 3:** Descriptive measures of the scores for the total and the dimensions from the QoPL-35 Questionnaire of 40 nurses working in ICU

Scale / Dimensions	Median	Quartile 1	Quartile 3	Minimum	Maximum
Total	213	190	240	152	288
Directive support	65	52	77	26	100
Workload	68	56	80	38	110
Intrinsic motivation	79	68	86	50	94
Perceived professional quality of life	5	4	8	2	10

Through the division by tertiles of the dimensions of the QoPL-35, it was established that only 40% of the participants perceive high directive support, 37.5% considered being exposed to high workload, and 70% has between medium and high intrinsic motivation. (Table 4).

**Table 4:** Division by tertiles of the dimensions from the QoPL-35 Questionnaire of 40 nurses working in ICU

Dimensions	Number	Percentage
Directive support		
Low	13	32.5
Medium	11	27.5
High	16	40
Workload		
Low	13	32.5
Medium	12	30
High	15	37.5
Intrinsic motivation		
Low	12	30
Medium	14	35
High	14	35

Through the Maslach Burnout Inventory instrument, it was determined that 22.5% of the nurses have high burnout syndrome, 65% medium, and 12.5% low. Emotional exhaustion affects 77.5% of the participants, 37.5% has low personal achievement, and only 15% manifests high degree of depersonalization. (Table 5). 

**Table 5:** Burnout syndrome according to dimensions and total from the Maslach Burnout Inventory instrument in 40 nurses working in ICU

Dimensions from the MBI	Number	Percentage
Low	5	12.5
Medium	26	65
High	9	22.5
Emotional exhaustion		
Low	9	22.5
Medium	23	57.5
High	8	20
Depersonalization		
Low	25	62.5
Medium	9	22.5
High	6	15
Personal achievement		
Low	15	37.5
Medium	14	35
High	11	27.5

Significant statistical association exists between some sociodemographic characteristics and the dimensions of the QoPL-35 questionnaire; thus, it is evident that intrinsic motivation is higher in participants with 1 to 2 economic dependents; nurses with > 10 years of professional practice in ICU perceive greater workload, professionals have greater intrinsic motivation and perceive higher directive support than the residents, however, they have a higher workload (Table 6). Likewise, nurses < 41 years of age have better perceived professional quality of life (independent question) than those from the other age groups (*p* = 0.002).

**Table 6:** Dimensions of the QoPL-35 Questionnaire according to sociodemographic characteristics in 40 nurses working in ICU

Characteristic	QoPL-35 Dimension	QoPL-35 Dimension	QoPL-35 Dimension	***p -* value**
	**Low *n* (%)**	**Medium *n* (%)**	**High *n* (%)**	
Economic dependents	Intrinsic motivation	Intrinsic motivation	Intrinsic motivation	0.045
0 (*n* = 16)	4 (25)	9 (56.3)	3 (18.7)	
1 (*n* = 14)	3 (21.4)	4 (28.6)	7 (50)	
2 (n = 6)	2 (33.3)	0	4 (66.7)	
3 or more *(n* = 4*)*	3 (75)	1 (25)	0	
Years of professional practice	Workload	Workload	Workload	0.034
≤ 5 (*n* = 23)	11 (47.8)	3 (13)	9 (39.2)	
6 - 10 (*n* = 6)	0	5 (83.3)	1 (16.7)	
11 - 15 (*n* = 4)	1 (25)	1 (25)	2 (50)	
≥ 16 (*n* = 7)	1 (14.3)	3 (42.8)	3 (42.8)	
Academic level	Intrinsic motivation	Intrinsic motivation	Intrinsic motivation	0.043
Professionals (*n* = 27)	9 (33.3)	6 (22.2)	12 (44.4)	
Residents (*n* = 13)	3 (23.1)	8 (61.5)	2 (15.4)	
Academic level	Workload	Workload	Workload	0.024
Professionals (*n* = 27)	5 (18.5)	10 (37)	12 (44.5)	
Residents (*n* = 13)	8 (61.5)	2 (15.4)	3 (23.1)	
Academic level	Directive support	Directive support	Directive support	0.025
Professionals (*n* = 27)	5 (18.5)	9 (33.3)	13 (48.2)	
Residents (*n* = 13)	8 (61.5)	2 (15.4)	3 (23.1)	

Medium and high BS prevalence is higher in participants > 41 years of age, with 11 or more years of professional practice and with regular professional quality of life; in contrast, younger nurses, less time of professional exercise, and good QoPL tend to have lower severity of the BS (Table 7). 

**Table 7:** Prevalence of the burnout syndrome according to sociodemographic characteristics in 40 nurses working in ICU

Characteristic	Burnout syndrome	Burnout syndrome	Burnout syndrome	***p -* value**
	Low	Medium	High	
Age (years)				0.048
≤ 30 (*n* = 15)	2 (13.3)	8 (53.3)	5 (33.3)	
31 to 40 (*n* = 15)	3 (20)	11 (73.3)	1 (6.7)	
41 to 50 (*n* = 4)	0	2 (50)	2 (50)	
≥ 51 (*n* = 6)	0	5 (83.3)	1 (16.7)	
Years of professional practice				0.038
≤ 5 (*n* = 23)	3 (13)	15 (65.2)	5 (21.7)	
6 - 10 (*n* = 6)	2 (33.3)	3 (50)	1 (16.7)	
11 - 15 (*n* = 4)	0	3 (75)	1 (25)	
≥ 16 (*n* = 7)	0	5 (71.4)	2 (28.6)	
Professional quality of life (QoPL)				0.040
Good (*n* = 12)	5 (41.7)	6 (50)	1 (8.3)	
Regular (*n* = 28)	0	20 (71.4)	8 (28.6)	
Poor (*n* = 0)	0	0	0	

Moreover, it was determined that significant relations exist among the dimensions that compose both instruments; lower directive support indicates higher emotional burnout (*p*=0.015) and with greater workload, lower personal achievement is perceived (*p*=0.009).

## Discussion

In the ICU, the nursing staff is exposed to high and sustained levels of stress, mental, physical, and emotional exigencies that predispose them to physical wear, job dissatisfaction, emotional burnout, low QoPL, and development of the BS with negative effect in their performance, health and quality of the care provided to patients.(5,10) This research evidences regular professional quality of life with the following behavior: high intrinsic motivation, medium workload and medium to low directive support, findings that agree with Canova-Barrios *et al.,*(3) and Fernández-Araque *et al.,*(11) a situation also found by Monroe *et al.,*(12) who also associated lack of directive support with the development of low QoPL, emotional exhaustion and job dissatisfaction in ICU.

The diagnosis of high BS has a prevalence of 22.5%, far surpassing the findings by Maticorena-Quevedo *et al.,*(13) and Salgado-Roa *et al.,*(2) with results of 2.1% and 10% in the nursing staff working in ICUs in Peru and Chile, respectively, findings also above those by Van der Heijden *et al.,*(14) who additionally concludes that a strong relationship exists between the prevalence of the BS and greater occurrence of adverse effects associated with care. Of the dimensions that make up the BS, emotional burnout has the highest influence and affects 77.5% of the participants. These results are equivalent to those by Vega *et al.,*(15) where emotional burnout was the principal determinant for the development of the BS and had a 65% prevalence; in the same way as Monsalve-Reyes *et al.,*(16) depersonalization has little presence in this study; however, 37.5% perceive low personal achievement; a condition associated with depression and anxiety product of a sense of stagnation and which is capable of triggering emotional burnout and conditioning the subsequent appearance of cynicism or dehumanized treatment.(17)

Significant statistical associations exist between the sociodemographic characteristics and the dimensions that structure the QoPL-35; thus, similar to the findings by Fernandez-Araque *et al.,*(11) the staff < 41 years of age manifests lower perceived professional quality of life than those of higher age and the staff with more time of professional exercise perceives greater workload. As in Jang *et al.,*(18) professionals with degrees in intensive care perceive better directive support and have better intrinsic motivation than the residents; in their research, this behavior was attributed to greater experience, confidence, skills and communication abilities acquired during the professionalization. At the same time, and in keeping with the study by Galindo *et al.,*(19) professionals perceive greater workload than the students, associating this result with the extra responsibility of surveillance and advice to the students. Finally, as in Lee *et al.,*(20) the participants with economic dependents reported higher intrinsic motivation.

This research demonstrated a significant statistical relation among the severity of the BS, age, and time of professional practice; participants in the age group of 41 or more years and with > 10 years of professional practice have a higher prevalence of medium and high burnout syndrome, these results differ from Mefoh *et al.,*(21) and Molero-Jurado *et al.,*(22) who found higher severity of the BS in young individuals who have less protection mechanisms against stress and are more prone to suffering its negative effects. In turn, as described by Roberts *et al.,*(23) and Kwak *et al.,*(24) a lower QoPL indicates higher presence of emotional burnout, job dissatisfaction, and high BS.

As a limitation, it must be stated that this was a study in a single hospital center and of cross-sectional design, making it difficult to extrapolate the results to other ICUs in the country. However, these results offer valuable and relevant information from the administrative point of view upon identifying points susceptible to intervention to improve the QoPL and diminish the BS prevalence and avoid its unwanted effects.

This research permits concluding that the QoPL of the nursing staff in the ICU studied is regular, high intrinsic motivation of the staff exists, workload is high, and 60% of the nurses perceive that directive support is from low to medium. The different dimensions that compose the QoPL have different behaviors related with age, time of professional exercise in ICU, number of economic dependents, and academic level. The high BS prevalence is significantly above that described in other research from the region; emotional exhaustion affects 77.5% of the participants and 37.5% has low personal achievement, however, depersonalization remains in low levels. It was demonstrated that the regular level of QoPL is related with higher severity of the BS.

According to the authors’ knowledge, when conducting this study, no evidence was documented on the professional quality of life of the nursing staff in an ICU in Venezuela, which is why their behavior and association with the BS was totally unknown.
